# Supplementary material for: Investigating water movements around a shallow shipwreck in Big Tub Harbour of Lake Huron: Implications for managing and preserving underwater shipwrecks
Source: PLoS One. 2023 Jan 3;18(1):e0279862. doi: 10.1371/journal.pone.0279862 (PMC9810187; doi:10.1371/journal.pone.0279862)
Supplement: Attachment — Submitted filename: Response to Reviewers.docx [file pone.0279862.s006.docx]

**Response to review comments on manuscript reference PONE-D-21-24254, Investigating the water movements around a shallow shipwreck in Big Tub Harbour of Lake Huron: implications for managing underwater shipwrecks (Flood et al.)**

We would like to thank both reviewers and the editor for their insightful comments. We understand the effort required to read a manuscript in depth and come up with constructive feedback and appreciate the time they dedicated to helping us improve our paper. We have taken into consideration all the comments and have made meaningful changes to our original document. We agree with most of the comments and have enacted almost all the recommended changes. This document tabulates all the received comments (in black text), along with our responses and the revisions we have made to the manuscript (in green text). We have endeavored to answer all the questions posed, and where we agree with the Reviewers’ suggestions, we have clearly outlined what changes we have made. On the rare occasion where we did not implement the Reviewers’ suggestions, we have clearly stated our reasoning. Because page and line numbers changed during the revision process, we note the page/line number of the text from the original manuscript, unless otherwise noted. We believe these revisions have resulted in substantial, meaningful improvements to our paper and are grateful for the opportunity to resubmit to PLOS ONE.

Response to editor’s comments

1. Please ensure that your manuscript meets PLOS ONE's style requirements, including those for file naming. The PLOS ONE style templates can be found at

Reference format has been revised to match PLOS ONE guidelines.

Title, author, and affiliation styles have been formatted to conform with PLOS ONE style guide.

The manuscript body has been formatted in conformance with PLOS ONE formatting guidelines.

File names have been changed to match PLOS ONE file naming convention.

All figures have been corrected to ensure they meet PLOS requirements via the PACE digital diagnostic tool.

2. We note that Figures 1-3 in your submission contain [map/satellite] images which may be copyrighted.

3. Please include your tables as part of your main manuscript and remove the individual files. Please note that supplementary tables (should remain/ be uploaded) as separate ""supporting information"" files"""

Tables and table captions have been embedded in the manuscript document, and individual table files have been removed from the editorial manager.

4. Please include captions for your Supporting Information files at the end of your manuscript, and update any in-text citations to match accordingly. Please see our Supporting Information guidelines for more information: <http://journals.plos.org/plosone/s/supporting-information>.

Captions for Supporting Information files have been included at the end of the manuscript document and all in-text citations have been updated.

Response to reviewers’ comments

Reviewer #1:

- We prefer if you use the third person singular, instead of the first person singular or plural (e.g. "we").

We are aware that some scientific writing uses a very formal approach with the third person singular, but it we feel it overly dry. Indeed, it is very common to use first person plural, especially when there is a narrative about field work - "we" were certainly out there doing this work! In another article documenting field work, that is published in PLOS-One (<https://journals.plos.org/plosone/article?id=10.1371/journal.pone.0057444>) “we”  was used 25 times without comment from the editor. Unless this is PLOS-ONE policy, we would like to stick with this style.

- More suitable title should be selected for the article.

We appreciate this suggestion and have revised the title, more thoroughly encapsulating the essence of the study, allowing potential readers to understand what the article is about by just reading the title.

Original title:

*“**Investigating the water movements around a shallow shipwreck in Big Tub Harbour of Lake Huron: implications for managing underwater shipwrecks.”*

Revised title:

*“Investigating water movements around a shallow shipwreck in Big Tub Harbour of Lake Huron: implications for managing and preserving underwater shipwrecks”*

- The abstract should state briefly the purpose of the research, the principal results and major conclusions. An abstract is often presented separately from the article, so it must be able to stand alone.

We agree that the abstract should, on its own, encompass the purpose, methods, and major findings of the study. Based on the helpful comments from both reviewers, we have revised the abstract. We have incorporated the need for and purpose of the research, further highlighted the major conclusions and scientific value, and included the study limitations.

- It is suggested to present the structure of the article at the end of the introduction.

We agree that it is important for scientific papers to have a well-defined and easy to follow structure. As such, our paper follows the typical IMRAD format for scientific articles, which the reader is expected to be familiar with. Nonetheless, we agree that outlining the structure of the paper can be very useful for the reader and have added a brief paragraph to that effect at the end of the introduction.

Original text:

NA

New text (starting on line 141 of original manuscript):

*“The article is structured as follows: the study site, field measurements, data processing and analysis techniques are presented in the Methods section, followed by a summary of the observations and analysis in the Data and Results section. A discussion of the implications and limitations of our findings are presented in the Discussion section. A brief summary of the study and our findings are provided in the Conclusion section.”*

- The major defect of this study is the debate or Argument is not clear stated in the introduction session. Hence, the contribution is weak in this manuscript. I would suggest the author to enhance your theoretical discussion and arrives your debate or argument.

This article aims to determine if ship use by tourism is destabilizing the most iconic shipwreck in Canada. In the introduction, at the end of first paragraph (starting at line 63 of original manuscript) and beginning of the second we have added the following text to make this clear:

*One challenge for conservation authorities is how to manage access to such shipwreck sites – in particular making sure that there are no strong water currents from tourist vessels that could damage these underwater structures. With the increasing interest in such shipwrecks in the Great Lakes it is important to understand how currents induced by tourist vessels compare with the intrinsic variability of natural currents.*

*Fathom Five National Marine Park (FFNMP), Lake Huron, Canada is one such protected area facing the challenge of managing tourist access with the preservation of culturally important shipwrecks (Fig. 1a).*

- More suitable title should be selected for the table 1 instead of “Summery of instruments deployed in Big Tub Harbour in 2015.”

We appreciate having this generic table caption brought to our attention, as we feel figure and table captions should describe the information they are displaying. We have therefore revised the Table 1 caption to be more descriptive.

Original title:

*“Summery of instruments deployed in Big Tub Harbour in 2015.”*

Revised Title:

*“**Location, measured water quality parameter, and observation frequency of instruments deployed in Big Tub Harbour, 2015”*

- It is suggested to compare the results of the present research with some similar studies which is done before.

We thank the reviewer for this comment and agree that it is important to compare our current study with similar research, positioning our new work and findings within the larger body of knowledge. This allows the reader to more easily understand the contributions and significance of the current study. To this effect we have added a paragraph to the discussion section, discussing similar work by Fernandez-Montblanc in energetic tidal systems.

Original text:

NA

New text (starting at line 721 of original manuscript):

“*Investigations of shallow-water shipwrecks in marine settings with energetic tidal systems have shown that the accretion and erosion processes that lead to scour rely on a complex inter-play of wave-induced currents and steady flow, seasonal variability in wave energy, and site conditions [19, 33]. Our findings suggest that these same processes likely influence scour around shipwrecks in lower-energy freshwater lake systems.”*

- It is suggested to add articles entitled “Nazarnia et al. A Systematic Review of Civil and Environmental Infrastructures for Coastal Adaptation to Sea Level Rise”, “Gholami & Baharlouii. Monitoring Long-term Mangrove Shoreline Changes along the Northern Coasts of the Persian Gulf and the Oman Sea” and “Cham et al. An Analysis of Shoreline Changes Using Combined Multitemporal Remote Sensing and Digital Evaluation Model” to the literature review.

We appreciate the literature review suggestions, as we believe it is important to thoroughly review and include relevant research to describe the current state of research in the field and the need for the current study. We have therefore included a discussion of how novel remote sensing and modeling techniques developed by Cham et al. (2020) could be applied to aid the investigation of temporal scour-dynamics in our system.

Original text:

NA

New text (starting at line 733 of original manuscript):

“*Multitemporal remote sensing and digital evaluation modeling following Cham et al. [33] could also be applied to investigate the temporal evolution of the scour around the Sweepstakes, both intra- and inter-seasonally, providing details on degradation rates to inform management decisions.”*

- Page 14: the following paragraph is unclear, so please reorganize that:
“We compare the temperature time series acquired in the direct vicinity of the Sweepstakes and at the mouth of the harbour to see if there are any intrusive cold gravity flows in Big Tub Harbour induced by the upwelling of cold waters in Lake Huron. The upwelling events are identified as a drop in water temperature by 5-8 0C in the space of few hours.”

We thank the reviewer for bringing this issue to our attention – it is important that scientific publications be clear and understandable to facilitate knowledge transfer and dissemination. As such, we have revised the above paragraph.

Original text (lines 289-292 of original manuscript):

*“We compare the temperature time series acquired in the direct vicinity of the Sweepstakes and at the mouth of the harbour to see if there are any intrusive cold gravity flows in Big Tub Harbour induced by the upwelling of cold waters in Lake Huron. The upwelling events are identified as a drop in water temperature by 5-8 0C in the space of few hours.”*

Revised text:

*“Cold water intrusions could be a source of energy for scouring. These could be caused by upwelling of cold water from Lake Huron into Big Tub Harbour. To determine if such intrusive currents are present, we monitored water temperatures at mouth of harbour and near the Sweepstakes. The cold intrusive events are identified as a drop in water temperature by 5-8°C in the space of few hours.”*

- Much more explanations and interpretations must be added for the Results, which are not enough.

We thank the reviewer for their suggestion. We have added new topic sentences to nearly every paragraph in the Results section to clarify the subject of each section.

- Please make sure your conclusions' section underscore the scientific value added of your paper, and/or the applicability of your findings/results, as indicated previously. Please revise your conclusion part into more details. Basically, you should enhance your contributions, limitations, underscore the scientific value added of your paper, and/or the applicability of your findings/results and future study in this session.

We appreciate the reviewer’s comment and suggestion as it is important to us that the scientific value of our study, as well as the main findings and limitations are clearly stated. We have therefore revised the conclusions to clarify the scientific value of our findings. This has been achieved by simplifying some of the text and adding clear statements to highlight the findings and their scientific value. For example, lines 738 – 743 of the original manuscript,

*“Field observations suggest that the circulation induced by internal gravity waves derived from upwelling did not increase current speeds in the vicinity of the shipwreck, suggesting currents derived from internal gravity waves are unlikely to be a significant cause of erosion and scour around the Sweepstakes during summer. As observed, there does not appear to be a meaningful difference in water currents between when tour boats are present or absent.”*

were changed to,

*‘Our extensive field observations during the peak summer tourist season suggest that there does not appear to be a meaningful difference in water currents around the Sweepstakes between times when tour boats are present or absent, and that in general water currents are weak. This is favourable to the long-term preservation of the Sweepstake shipwreck – one of the most iconic shipwrecks in Canada.”*

Text was also added to reiterate the limitations, and possible future research directions (added at line 756 of original manuscript):

*“Rather the scour is probably caused by winter storms, which should be a focus of future studies.”*

-DOI of the references must be added (you can use “http:/crossref.org/”).

In our experience DOI are added at the proof stage and thus have not added to the submitted manuscript.

Reviewer #2:

The authors collected a wide variety of field measurements at multiple locations and times and aimed to assess the potential impact of human-derived water movements on shipwrecks. They quantify various parameters of human-derived currents and of natural water currents. It is quite laborious to well collect and analyze such a rich data, while the logical structure of the context is clear. In many instances, the tourism developments impact the conservation and preservation of the natural environment and cultural heritages. Scientific evidence and surveillance regarding to the damaging effect would conduce to develop their mutually beneficial module. This study gave real maneuvers on acquiring observation and objective inference in a scientific way. To facilitate practical reference, some suggestions proposed.

Although the results driven from various measurements were summarized in tables and elaborated in content, no inferential statistical analyses were performed. According to the description on data collection, repeated measurement analysis or time series analysis could be used. Other comparable methods are also usable, as long as their applicability is reasonable. At least, 95% confidence intervals for point estimates, e.g., in tables, are recommended to be supplemented.

We thank the reviewer for their thoughtful suggestion. However, we do not feel the use of inferential statistics is the most appropriate in our study. The main point of this research is not to estimate (predict) the currents (thus not warranting inferential statistics), but to evaluate the existing (available) distribution under different circumstances. For example, if we were to try and determine the 95% confidence interval (CI) for current speed when boats were present and absent, we would have to assume we have a sample that accurately represents a population, and the 95% CI helps us constrain, or describe our estimate of the mean value of the population, based on our limited sample size. This is not the case with current speed – we are not trying to estimate the ‘true’ current speed, as in reality it is constantly varying due to a combination of simultaneous processes. What we would like to do is describe the range of observed current speeds under different scenarios (e.g., boats present or absent, or during upwelling events and non-upwelling times). To do this we have included descriptive statistics, which are more appropriate for our datasets and our analyses. For example, in Table 2 which tabulates water current speeds during upwelling and non-upwelling times, we have included the mean current speed, as well as the standard deviation to provide a measure of the spread or variability of the data and the 90^th^ percentile, to provide a measure of the fastest current speeds which are of particular interest, since it is these energetic currents that are most capable of causing scour. To provide an additional statistical measure and to describe our observations more fully, we have included the current speeds at the 5^th^, 25^th^, 75^th^, and 95^th^ percentiles for observed currents during upwelling and non-upwelling times (Table 2) and when boats are present or absent (Table 3).

In addition, condensed statements regarding the limitations mentioned in discussion and conclusions should be added in abstract.

We appreciate the suggestion to add study limitations to the abstract, as we agree it is important to highlight these in the abstract, which provides a more complete picture of the study and its findings in a condensed, easy to access format. In addition to other revisions we made to the abstract to clarify the purpose and need for the current research, and to highlight the major conclusions, we have also included the pertinent study limitations in the abstract (at line 31 of original manuscript):

*“Scour is most likely caused by energetic winter storms, which should be a focus of future studies.”*
