# Supplementary material for: Investigating water movements around a shallow shipwreck in Big Tub Harbour of Lake Huron: Implications for managing and preserving underwater shipwrecks
Source: PLoS One. 2023 Jan 3;18(1):e0279862. doi: 10.1371/journal.pone.0279862 (PMC9810187; doi:10.1371/journal.pone.0279862)
Supplement: Attachment — Submitted filename: Response to Reviewers.docx [file pone.0279862.s007.docx]

Investigating water movements around a shallow shipwreck in Big Tub Harbour of Lake Huron: implications for managing and preserving underwater shipwrecks.

Bryan Flood, Lakshika Girihagama, Mathew G. Wells, Reza Valipour, Patricia Semcesen, and Scott Parker.

PONE-D-21-24254R1

Responses to editor and reviewer comments, second round of revisions

We thank the editor and reviewers for their helpful second round of comments. We have included all the suggestions in the revised text, and detail the changes below. We hope that with this second round of revisions that the paper can now be published in PLOS-ONE.

Editor and reviewer comments are documented below in black text, while responses to those comments and the changes we’ve made to the manuscript are in red text.

Journal Requirements

References have been reviewed to ensure none have been redacted. Additionally, due to the addition of several new references as a result of the revisions, the reference numbers have been reviewed as revised as required. The new references are:

22.	Hlevca, B., Cooke, S.J., Midwood, J.D., Doka, S.E., Portiss, R. and Wells, M.G. Characterisation of water temperature variability within a harbour connected to a large lake. Journal of Great Lakes Research. 2015; 41(4), pp.1010-1023.

23.	Hlevca, B., Wells, M. G., Cruz Font, L., Doka, S. E., Portiss, R., St. John, M., & Cooke, S. J. Water circulation in Toronto Harbour. Aquatic Ecosystem Health & Management. 2018; 21(3), 234-244.

24.	Girihagama, L., Howell, E. T., Li, J., & Wells, M. G. Physical Circulation in the Coastal Zone of a Large Lake Controls the Benthic Biological Distribution. Water Resources Research. 2022; 58(3), e2021WR030412.

33.	Boegman, L. Currents in stratified water bodies: Internal waves. Encyclopedia of inland waters, Elsevier, 2022, 510-524.

34.	Peeters, F. and Kipfer, R. (2022) Currents in Stratified Water Bodies 1: Density-Driven Flows. Encyclopedia of Inland Waters, Elsevier, 2022. 525-534

Editor Comments

A sentence on application and understanding of water movements in other similar ecosystems like Big Tub Harbour of Lake Huron would further benefit the paper.

Thank you for the valuable suggestion. We have included a couple sentences to highlighted a couple other systems with similar dynamics to add additional context.

Original sentence:

N/A

Revised sentence (lines 134-140 revised manuscript):

*There are numerous deep embayments in the Great Lakes with similar dynamics to this study. For instance, the water flows in the Toronto Harbour (in Lake Ontario) are analogous to the flows we measured in big Tub Harbour, in that both are protected embayments connected to great lakes. In particular, Toronto harbours experiences periodic water currents up to 10 cm/s driven by 1-hour surface seiches, as well as periodic intrusions of cold stratified water [22, 23]. Similar flows are also seen in the many hundreds of embayments that exist behind the coastal archipelago along the eastern shore of Georgian Bay [24].*

This resulted in including 3 additional references:

Hlevca, B., Cooke, S.J., Midwood, J.D., Doka, S.E., Portiss, R. and Wells, M.G. Characterisation of water temperature variability within a harbour connected to a large lake. Journal of Great Lakes Research. 2015; 41(4), pp.1010-1023.

Hlevca, B., Wells, M. G., Cruz Font, L., Doka, S. E., Portiss, R., St. John, M., & Cooke, S. J. Water circulation in Toronto Harbour. Aquatic Ecosystem Health & Management. 2018; 21(3), 234-244.

Girihagama, L., Howell, E. T., Li, J., & Wells, M. G. Physical Circulation in the Coastal Zone of a Large Lake Controls the Benthic Biological Distribution. Water Resources Research. 2022; 58(3), e2021WR030412.

Reviewer #2: The author did appropriate revision partially and provided explanations on revisions which did not comply to the modification suggestions. This study did contribute abundant evidence and demonstrated an objective assessment manoeuvre for underwater antiquities deteriorated by human activities. I accept the authors' way and their explanations on revisions.

We appreciate the kind words of the reviewer.

Reviewer #3: I congratulate the authors for considering and efficiently revising the manuscript. The manuscript now seems to be of publishable form.

We appreciate the kind words of the reviewer.

Reviewer #4: A few of my concerns are as follows:
 1. Justification or interpretation should be limited to the discussion section, the glimpses of which could be seen in the results sections.

We appreciate having this issue brought to our attention and have removed the following sentences to avoid having discussion statements in the results section.

Original sentence (lines 481-482 original manuscript):

*These differences, however, are small (0.1 – 0.4 cm s-1) and unlikely to influence resuspension potential.*

Revised sentence:

N/A

Original sentence (lines 589-601 original manuscript):

*This suggests evening and nighttime peaks in pressure fluctuations were likely due to wind forcing and/or recreational divers.*

Revised sentence:

N/A

2. Same goes for the conclusion, which has some results mentioned in it, instead of only concluding statements.

We agree that it is important to adhere to the well-established IMRAD scientific article format and have revised our conclusion section to remove specific results. The sentence on lines 763-765 was removed. We feel this is acceptable, as the following sentence summarizes the results specifically mentioned in lines 763-765. The conclusion section now focuses entirely on concluding remarks without referencing specific results.

Original sentence (lines 763-765 original manuscript):

*Analysis of bottom currents during presence and absence of tour boats show that while the 90^th^ percentile of current speeds were 1.1 – 1.3 cm s^-1^ faster at the side of the wreck when tour boats were present, they were 0.8 cm s^-1^ slower at the prow.*

Revised sentence:

N/A

3. Line no: 138 "affect" needs to change with "effect".

We appreciate having this spelling/grammar error brought to our attention. We have changed ‘affect’ to ‘effect’ on line 138 (line 145 of revised manuscript)

4. Line no: 725-728 needs improvement. The statement repeats the citation of Boyce, three times in a single sentence, while making it unclear if the data mentioned, belongs to Boyce, or your study, specifically because of the use of "our study" in between the sentence.

We thank the reviewer for noting this challenging sentence. We agree that the way it was originally worded resulted in ambiguities as to which study the reported values belonged to. We have revised this by splitting it into two sentences and clarifying which values belong to which study.

Original sentence (line 725-728 original manuscript):

*Despite a notable increase in vessel size (~40% larger) and frequency of use, our study found that the maximum velocities at same location by Boyce [18] on the port side of wreck were at most ~5 cm s^-1^, and always much less than the maximum values of 12 cm s^-1^ observed by Boyce [18] when tour vessels were operated aggressively, and the isolated peak currents of 11.2 cm s^-1^ and 17 cm s ^-1^ Boyce [18] observed in 1993 and 1994, respectively.*

Revised sentence (line 727-732 revised manuscript):

*Despite a notable increase in vessel size (~40% larger) and frequency of use, the current study found that the maximum velocities on the port side of wreck (same observation location as Boyce [18]) were at most ~5 cm s^-1^. They were always much less than both the maximum values of 12 cm s^-1^ observed by Boyce [18] when tour vessels were operated aggressively, and the isolated peak currents of 11.2 cm s^-1^ and 17 cm s ^-1^ Boyce [18] observed in 1993 and 1994, respectively.*

Reviewer #5: It is commendable that the authors collected a range of field measurements in various locations throughout the study site. The hypothesis for this journal also seems to make sense. I appreciate the authors' efforts; however, I would want to make a few suggestions.
 • The authors have not been precise; for example, lines 547 to 552 contain numerous repetitions of the same statement.

We appreciate having this repetition brought to our attention. We have improved the precision of the manuscript by removing repetitive statements in the noted paragraph resulting in both more concise and clear writing.

The sentence on lines 542-544 was revised to remove ‘as based on video footage’ as this is clarified later in the paragraph and does not require repeating.

Original sentence (lines 542-544 original manuscript):

*The effect of tour boat propeller wash on water currents was evaluated by comparing the bottom water currents in the immediate vicinity of the Sweepstakes when boats were present and when they were not (as based on video footage).*

Revised sentence (lines 548-550 revised manuscript):

*The effect of tour boat propeller wash on water currents was evaluated by comparing the bottom water currents in the immediate vicinity of the Sweepstakes when boats were present and when they were not.*

Furthermore, we revised the sentence on lines 547-550 to remove ‘compared to when they are not present’ as this alternate scenario is obvious based on the previous sentences and does not need to be articulated.

Original sentence (lines 547-550 original manuscript):

*If, however, water currents are not significantly faster during times when boats are present, compared to when they are not present, it would imply that the tour boats do not significantly disturb the water more than natural variability does.*

Revised sentence (lines 553-555 revised manuscript:

*If, however, water currents are not significantly faster during times when boats are present, it would imply that the tour boats do not significantly disturb the water more than natural variability does.*

• Also, I would like recommend some more minor adjustments like: -
 i. Line 41 (keyword section)- There are two phrases here, namely "shallow shipwrecks" and "water movement." These phrases may be substituted with alternative phrases as they are already present in line 1, i.e., title section.

We appreciate the recommendation, and agree that phrases contained in the paper’s title do not need to be repeated as keywords. We have removed both ‘shallow shipwrecks’ and ‘water movements’ from the keyword section and have included ‘shallow embayment’ which will assist readers looking for hydrodynamic studies conducted in similar environments (i.e., shallow embayments).

ii. Line 100- “A ring of erosion is visible around the Sweepstake where no aquatic vegetation (Chara sp.)”, why is Chara sp. listed in brackets while it is said that there is no vegetation present?

Upon review, we agree that the inclusion of the absent aquatic plant species is confusing. It was originally included to highlight what the dominant species in the vicinity was, and that it was not present in the erosion ring around the Sweepstakes. We have removed it from the revised manuscript, and changed ‘are’ to ‘is’ to be grammatically consistent.

Original sentence (line 100 original manuscript):

*A ring of erosion is visible around the Sweepstake where no aquatic vegetation (Chara sp.) are growing and sand is exposed.*

Revised sentence (lines 99-100 revised manuscript):

*A ring of erosion is visible around the Sweepstake where no aquatic vegetation is growing and sand is exposed.*

iii. Line 105- full form/detailed about ADP should be mentioned.

We have amended the manuscript to include the full form of the ‘ADP’ acronym on line 105.

Original sentence (line 105 original manuscript):

*(f) An underwater photograph of the prow of the Sweepstakes, the yellow ADP is visible in background on the bed.*

Revised sentence (lines 104-105 revised manuscript)

*(f) An underwater photograph of the prow of the Sweepstakes, the yellow acoustic Doppler profiler (ADP) is visible in background on the bed.*

iv. Line 427 to 428- “Stratified water bodies often have strong currents associated with internal waves that can be a strong source of oscillating currents” any citations would strengthen this assertion.

We now have included citations to two review articles that appear in the new 2022 edition of the Encyclopedia of Inland Waters. Professors Leon Boegman and Frank Peeters are world experts on currents induced by stratified internal waves in lakes. Note that the new line numbering (revised manuscript) for the sentence in question is 434-435.

Boegman, L. Currents in stratified water bodies: Internal waves. Encyclopedia of inland waters, Elsevier, 2022, 510-524.

Peeters, F. and Kipfer, R. (2022) Currents in Stratified Water Bodies 1: Density-Driven Flows. Encyclopedia of Inland Waters, Elsevier, 2022. 525-534

v. Line 465- “event or period” specific phrase can be maintained.

We appreciate having this terminology concern brought to our attention. We feel that while similar, ‘event’ and ‘period’ have subtle differences in meaning and application, and are used throughout the manuscript in different contexts. As such, we feel it is important to identify both terms here to avoid ambiguity throughout the manuscript.

vi. Line 469- “variability or maximum” specific phrase can be maintained.

We appreciate having this terminology concern brought to our attention. In this case, we are referencing two different variables – maximum velocity and velocity variability. As such, it is necessary to include both. We have addressed this confusion by re-wording the sentence to remove ambiguity.

Original sentence (line 469 original manuscript)

*However, if the variability or maximum velocities do not change, then the effect of internal gravity waves on the flows is minimal.*

Revised sentence (lines 476-478 revised manuscript):

*However, if the maximum velocities or velocity variability does not change, then the effect of internal gravity waves on the flows is minimal.*

**Other revisions**

Line 771-773 (original manuscript): ‘to’ was added to sentence to correct grammar mistake.

Original sentence (lines 771-773 original manuscript):

*Furthermore, the assessment of videos indicate that the boat induced pressure perturbations, instantaneous velocities, and induced dynamic forces on the shipwreck are unlikely drive resuspension.*

Revised sentence (lines 773-775 revised manuscript):

*Furthermore, the assessment of videos indicate that the boat induced pressure perturbations, instantaneous velocities, and induced dynamic forces on the shipwreck are unlikely to drive resuspension.*
